# Experimental Shifts in Intraclutch Egg Color Variation Do Not Affect Egg Rejection in a Host of a Non-Egg-Mimetic Avian Brood Parasite

**DOI:** 10.1371/journal.pone.0121213

**Published:** 2015-04-01

**Authors:** Rebecca Croston, Mark E. Hauber

**Affiliations:** 1 Ecology, Evolutionary Biology, and Behavior Subprogram in Biology, The Graduate Center of the City University of New York, New York, New York, United States of America; 2 Department of Psychology, Hunter College and The Graduate Center of the City University of New York, New York, New York, United States of America; Universidad de Granada, SPAIN

## Abstract

Avian brood parasites lay their eggs in the nests of other birds, and impose the costs associated with rearing parasitic young onto these hosts. Many hosts of brood parasites defend against parasitism by removing foreign eggs from the nest. In systems where parasitic eggs mimic host eggs in coloration and patterning, extensive intraclutch variation in egg appearances may impair the host’s ability to recognize and reject parasitic eggs, but experimental investigation of this effect has produced conflicting results. The cognitive mechanism by which hosts recognize parasitic eggs may vary across brood parasite hosts, and this may explain variation in experimental outcome across studies investigating egg rejection in hosts of egg-mimicking brood parasites. In contrast, for hosts of non-egg-mimetic parasites, intraclutch egg color variation is not predicted to co-vary with foreign egg rejection, irrespective of cognitive mechanism. Here we tested for effects of intraclutch egg color variation in a host of nonmimetic brood parasite by manipulating egg color in American robins (*Turdus migratorius*), hosts of brown-headed cowbirds (*Molothrus ater*). We recorded robins’ behavioral responses to simulated cowbird parasitism in nests where color variation was artificially enhanced or reduced. We also quantified egg color variation within and between unmanipulated robin clutches as perceived by robins themselves using spectrophotometric measures and avian visual modeling. In unmanipulated nests, egg color varied more between than within robin clutches. As predicted, however, manipulation of color variation did not affect rejection rates. Overall, our results best support the scenario wherein egg rejection is the outcome of selective pressure by a nonmimetic brood parasite, because robins are efficient rejecters of foreign eggs, irrespective of the color variation within their own clutch.

## Introduction

Hosts of brood parasitic birds face fitness costs associated with the rearing of genetically unrelated parasitic offspring [1]. Many hosts, across widely divergent brood parasite-host systems, have evolved defenses which either decrease the chances of being parasitized, or reduce the costs incurred as a result of parasitism [2, 3]. These defenses range from aggressive responses to adult brood parasites near the nests [4, 5] through foreign egg rejection [6, 7] to the rejection of brood parasitic chicks and fledglings (reviewed in [8]).

Recognition and removal of parasitic eggs from the nest is the most common host defense against parasitism [8,9]. Egg ejection, however, is an imperfect defense, and can itself lead to fitness losses for hosts through misrecognition and–rejection (i.e., recognition errors), or accidental damage to the hosts own eggs (i.e., rejection costs) [10,11]. Because of these costs, evolutionary theory predicts that hosts involved in an arms race with brood parasites will be under selective pressure to avoid recognition and rejection errors [12]. For some brood parasites, this results in laying of eggs mimicking host eggs in appearance (mimetic eggs [13]), while others lay eggs that do not appear to mimic those of their hosts (non-mimetic eggs [14], but see [15]). Egg mimicry is unlikely to evolve in systems where hosts do not reject parasitic eggs (i.e. no selective pressure toward mimicry), where parasites exploit a wide range of hosts with divergent egg phenotypes, or where there is evolutionary lag between parasites and hosts [9].

For hosts of mimetic-egg laying brood parasites, there are at least two strategies toward reducing the likelihood of recognition errors. A parasitized species can evolve towards 1) reduced within-clutch (intraclutch) variation in egg appearance (color and maculation), and/or 2) egg appearance unlike the parasitic eggs (e.g. [16,17]), effectively increasing between-clutch (interclutch) variability [17–22]. One or both of these patterns in clutch variation has been observed in many host species of the common cuckoo (*Cuculus canorus*, [20,21,23–25] but see [26]) and Diederik cuckoo (*Chrysococcyx caprius*, [22]), as well as in rejecters of intraspecific (functionally mimetic) parasitic eggs [27]. Many other observational, and a handful of experimental studies have, however, found inconsistent support for these patterns [21,28–32], and thus the literature as a whole is equivocal as to what extent brood parasitism and egg recognition fuel or limit the evolutionary trajectories of variation in both intra- and intraclutch egg color variability (Tables 1, 2).

**Table 1:** Summary of published studies using observational tests of the relationship between intraclutch egg appearance variability and rejection rate by hosts of obligate brood parasitic birds.^a^

Parasite	Host	Parasite Mim./Nonmim.	Correlation intra-	Correlation inter-	Reference
*Cuculus canorus*	Various	Mim.	None	Positive	[20]
*Cuculus canorus*	Various	Mim.	Negative	Positive	[23]
*Cuculus canorus*	*Acrocephalus arundinaceus*	Mim.	Positive	NA	[28]
*Cuculus canorus*	*Acrocephalus arundinaceus*	Mim.	None	Positive	[24] ^b^
*Cuculus canorus*	*Acrocephalus arundinaceus*	Mim.	Positive	NA	[32]
*Cuculus canorus*	*Acrocephalus scirpaceus*	Nonmim.	Negative	NA	[27]
*Cuculus canorus*	*Sylvia communis*	Nonmim.	None	NA	[29]
*Cuculus canorus*	*Anthus pratensis*	Mim.	Negative	NA	[25] ^b^
*Cuculus canorus*	*Lanius collurio*	Mim.	None	NA	[31]
*Cuculus pallidus*	*Lichenostomus penicillatus*	Mim.	None	Positive	[33]
*Clamator glandarius*	*Pica pica*	Mim.	Negative	NA	[34]
*Clamator glandarius*	*Pica pica*	Mim.	Positive	NA	[30]
*Chrysococcyx caprius*	*Ploceus cucullatus*	Mim.	Negative	Positive	[22]
*Molothrus ater*	Various	Nonmim.	None	None	[21]
*Molothrus ater*	Various	Mim.	None	None	[35]
*Molothrus ater*	Various	Nonmim.	None	None	[35]
*Molothrus ater*	*Quiscalus quiscula*	Nonmim.	Negative	NA	[36]

^a^ “Parasite Mim./Nonmim.” indicates whether natural parasitic eggs mimic those of hosts. “Correlation” indicates the direction of correlation (if any) between color variation within (“Correlation intra-”) and between (“Correlation inter-”) and the rejection rate of parasitic eggs.

^b^ Studies that compared inter- and intraclutch color variation between 2 populations, one in sympatry and one in allopatry with cuckoos. Positive correlation for interclutch color variation is derived from statistical difference between these two populations. Lack of correlation for intraclutch color variation is derived from lack of statistical difference between these two populations.

**Table 2:** Summary of published studies on egg rejection responses (relative to controls) to experimental brood parasitism, where the methodology included manipulations to increase intraclutch egg appearance variation.^a^

Parasite	Host	Parasite Mim./Non.	Exp. Mim./Nonmim.	Significant effect on rejection	Reference
*Cuculus canorus*	*Acrocephalus arundinaceus*	Mim.	Mim.	None	[26]
*Cuculus canorus*	*Acrocephalus arundinaceus*	Mim.	Nonmim.	Negative	[37]
*Cuculus canorus*	*Acrocephalus arundinaceus*	Mim.	Mim.	Negative	[37]
*Cuculus canorus*	*Acrocephalus arundinaceus*	Mim.	Both	Negative	[38] ^b^
*Anomalospiza imberbis*	*Prinia subflava*	Mim.	Mim.	Negative	[39]

^a^ “Parasite Mim./Nonmim.” indicates whether natural parasitic eggs mimic those of hosts. “Exp. Mim./Non.” indicates whether eggs used in artificial parasitism mimicked those of hosts. “Effect” indicates the induced change in the rate of rejection of experimental eggs.

^b^ To our knowledge, this is the only previous study to experimentally both increase and decrease intraclutch color variation.

For hosts of non-mimetic parasites, however, predictions about the effect of intra- and interclutch color variation are not yet well defined, and the effect of brood parasitism on intraclutch color variation is rarely addressed (but see [35]) for hosts of these types of brood parasites (Table 1, 2). We maintain and formalize (Table 3), that in the absence of egg mimicry, intraclutch color variation, and therefore also interclutch color variation, is relatively unconstrained by hosts’ need to recognize and reject foreign eggs, and is therefore free to vary in response to alternative selective pressures, physiological factors, and ecological factors including maternal condition, diet, and/or local predation pressures [40–43]. Alternatively, patterns of egg color variation may result from selective pressure from past inter- [44] or intraspecific [45] parasitism. While identifying the exact mechanism is beyond the scope of our study, we note that increased between-clutch egg color variation cannot be a definitive signature of selective pressure to reject eggs of a non-mimetic parasite, despite that the opposite trend more robustly indicates selective pressure to reject mimetic parasitic eggs. Similarly, experimentally increasing intraclutch color variation in these hosts is not predicted to affect rates of parasitic egg rejection (Table 3). Overall, hypotheses pertaining to the effects of parasitism on intraclutch color, and effects of intraclutch color on rejection rates, have gone largely untested in hosts of non-mimetic parasites, as there is little intraspecific variation in response to parasitism for these hosts [36]. Two studies to date have addressed the relationship between rejection rate and degree of intraclutch variation in a host of brown-headed cowbirds (*Molothrus ater*, a generalist brood parasite) host. Peer et al. [36] found that cowbird egg rejection was more likely when intraclutch variation was lower for common grackles (*Quiscalus quiscula*). However, in a subsequent comparison between acceptor versus rejector hosts laying blue and maculate beige eggs, respectively, intraclutch color variation did not vary with rejector status across either group [35].

**Table 3:** Summary of predictions for egg color variation and responses to experimental increase in intraclutch color variation based on different cognitive mechanisms underlying egg recognition, as a result of coevolution *per se* with mimetic versus nonmimetic brood parasites.^a^

	Discordancy			Template			Online self-reference		
	*Intra-*	*Inter-*	*Predicted effect*	*Intra-*	*Inter-*	*Predicted effect*	*Intra-*	*Inter-*	*Predicted effect*
**Mimetic parasite**	Decrease	Increase	Negative	Decrease	Increase	No effect	Decrease	Increase	Negative
**Nonmimetic parasite**	No effect	No effect	No effect	No effect	No effect	No effect	No effect	No effect	No effect

^a^“Predicted effect” represents the direction of the predicted effect of an experimental increase in intraclutch color variation on the probability of rejecting the parasitic egg.

Here we test for patterns of egg color variation within versus between unmanipulated non-mimetic brood parasite host clutches, and test for effects of experimentally increasing or decreasing intraclutch egg color variation on the likelihood of parasitic egg rejection. We combine observational and experimental approaches to analyze within and between-clutch color variation in a population of American robins (*Turdus migratorius*), a robust egg-rejecting host of obligate parasitic brown-headed cowbirds [2,46]. American robins are one of only ~26 hosts of extremely generalist [47,48] brown-headed cowbirds [49] to reject artificial and real cowbird eggs in up to 100% of trials where nests are experimentally parasitized [2,46]. We compare inter- and intraclutch color variation across the entire avian visual spectrum by combining spectrophotometric measures of egg color with statistical models describing the birds’ own spectral sensitivities [50]. Robins lay immaculate blue-green eggs, and cowbird eggs do not closely mimic those of their hosts [1] (but see [15]). Additionally, using artificial parasitism in combination with egg color manipulation, we experimentally test predictions associated with the role of intraclutch color variation in eliciting egg rejection (Table 3). Critically, we assess the effects of both experimentally increasing and decreasing intraclutch color variation but note that our theoretical considerations (Table 3) make a prediction of *no effect* on egg rejection rates for robins under either type of experimental treatment. If, however, robins evolved to reject foreign eggs due to selective pressures imposed by mimetic brood parasites (including intraspecific brood parasitism [45]), then we predict a decrease in egg rejection rates following experimental increases in intraclutch variability, if robins employ at least one of two known cognitive mechanisms used by rejecters to identify foreign eggs (Table 3).

## Materials and Methods

### Study site and nests

This study took place in and around Ithaca, Tompkins County, NY, USA from May-July in the breeding seasons 2010–2012. Nests were located through searches in and around buildings, bridges, barns, and clearing edges, especially in residential areas and farmland. Additional nests were located by enlisting the help of local residents using classified advertisements, signboards, and local internet communities.

### Egg color measurement and avian visual modeling

During the 2012 breeding season, we quantified eggshell color for complete, unmanipulated American robin clutches. All color measurements were taken on either the day of, or the first day following clutch completion, as blue-green color may fade over the course of incubation [51]. We quantified egg color across the entire avian visual spectrum by measuring spectral reflectance using a high resolution spectrometer with deuterium tungsten halogen light source and 455μm solarization-resistant shielded cable (Ocean Optics Jaz portable spectrometer with ultraviolet-visible (UV-VIS) light source, Ocean Optics Inc., Dunedin, FL, USA). Measurements were taken holding the fiber optic probe perpendicular to the egg surface. The spectrometer was calibrated using a Spectralon light reflectance standard (WS-1, Ocean Optics, Inc., which reflects > 95% of UV and visible light), and a black-box standard, which measures baseline noise in the spectrophotometer. The spectrometer was re-calibrated after measuring every third egg throughout sampling. The relative reflectance at each wavelength was calculated automatically with reference to the light and dark standards. To minimize measurement error, each egg was measured nine times, including three measurements each at the blunt pole, middle, and sharp pole, which were then averaged to yield one spectrum per egg.

To estimate degree of color variation both within and between unmanipulated robin clutches with respect to the spectral sensitivities of avian photoreceptors [52], we used the Vorobyev and Osorio [53] model for tetrachromatic vision in AVICOL v5 avian visual modeling software [54]. American robins are an ultraviolet-sensitive (UVS) species [55–57], but detailed spectral sensitivity data are not as yet available. We therefore extracted spectral sensitivity data for a congener, the European blackbird *T*. *merula*, from the published data in Hart et al. [58] using Vistametrix software (Vista Metrix 1.3, SkillCrest LLC) and ranging from 330–700nm. AVICOL requires sensitivity data ranging from 300–700 nm; we set photoreceptor absorbance for 300–330 nm to 0, *sensu* [59,60]. Relative cone densities were set to ultraviolet sensitive (UVS): 1.0, short-wavelength sensitive (SWS): 1.78, medium-wavelength sensitive (MWS): 2.21, long-wavelength sensitive (LWS): 1.96, and Weber fraction was set to 0.1 [58] *sensu* [59]. As the ability to discriminate different colors is influenced by environmental light [53] (but see [61]), we used published ambient light irradiance data for broken canopy forest [53], which may most closely simulate the variable forest-edge light environments in which many American robins nest, even when breeding in sub/urban sites [62].

Prior to analysis, we applied a correction to each egg spectra using triangular smoothing over 30 nanometers, available as a function within AVICOL, to attenuate the effect of spectrometer noise on the visual model. AVICOL extracts receptor catch quanta specific to each single-cone receptor type, and combines these with the known spectral sensitivities of the model taxon’s visual system (here *T*. *merula*) to quantify photoreceptor activity across the entire avian spectral sensitivity range and quantify birds’ abilities to distinguish between any two colors as the perceptual distance between spectra (ΔS), or JNDs (‘just noticeable differences’). By definition, JND values greater than 1.0 indicate a chromatic difference that is discriminable based on the published estimates of *T*. *merula* spectral sensitivities [63]. AVICOL can also be used to extract discriminability based on achromatic contrasts using the sum of the sensitivities of the MWS and LWS cones, as these are similar to the sensitivities of rods and principal double-cone cells in the avian retina [58].

For the sensory analysis, we extracted photoreceptor catches for each of the four avian single-cone receptors, and normalized these to 1 within the total reflectance of each egg, such that for each egg, we have calculated the proportion of total receptor catch that is attributable to each photoreceptor. We compared mean quantum catches for each photoreceptor across all nests using independent univariate ANOVAs, with the proportionate receptor catches (PrUVS, PrSWS, PrMWS, PrLWS) as response variables, and Nest ID as predictor. Likewise, we compared mean achromatic quantum catches among nests, repeating the above approach with achromatic quantum catch data for each egg, and comparing means across nests.

Finally, we compared discriminable difference values between eggs sharing a nest and eggs not sharing a nest as JNDs. To do this, we calculated JNDs differentiating each egg from every other egg in the data set. Then, to avoid pseudoreplication, we randomly selected among these paired comparisons such that each egg was included in the analysis only once. We compared mean within-nest JNDs to mean between-nest JNDs using univariate ANOVA, with type of comparison (within nest/between nest) as a predictor and JNDs as response.

### Egg rejection experiment

To experimentally test whether intraclutch color variation contributes to the ability of American robins to recognize and reject foreign eggs, we manipulated egg color within clutches, experimentally decreasing or increasing egg shell color variability [37]. We altered host eggs according to one of two treatments: in each, we removed eggs one at a time from nests, and painted each egg with one of two different blue/blue-green paints (acrylic, Artist’s Loft), chosen by spectrophotometric specifications of hue as determined by wavelength at peak reflectance, and of known [64], low (0–20% for cowbird-sized model eggs) rejection rates. Eggs were allowed to dry fully before being returned to the nest.

**Fig 1:**
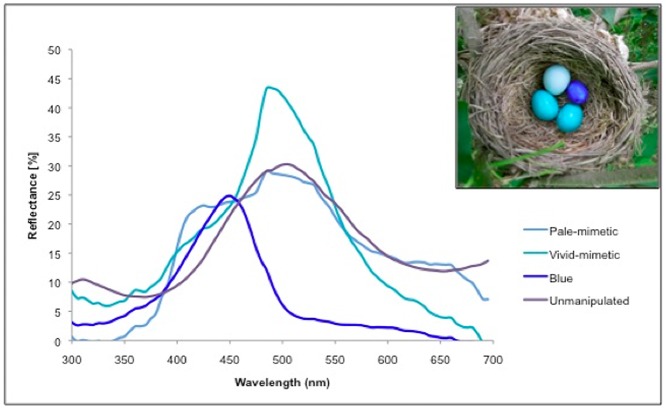
Representative egg color spectra, with experimentally manipulated nest (inset). Representative spectra showing each of the three colors used in the egg rejection experiment, in addition to natural American robin egg spectrum. Pale-mimetic and vivid-mimetic paints were used to manipulate the color of real robin eggs. Blue paint was used to color plaster-of-Paris model parasitic eggs. The unmanipulated spectrum represents the average spectrum of real robin eggs. Inset shows a representative nest with experimentally increased variation in egg color, showing two natural robin eggs painted with vivid-mimetic paint, one painted with pale-mimetic paint, and one blue model egg.

In order to increase the amount of color variation within a clutch (increased color variation treatment; IV), we painted two eggs with either pale robin-mimetic or vivid robin-mimetic paint (Fig. 1) at random, and the third egg was painted the second blue-green shade (Fig. 1; see also inset). The second treatment group consisted of nests where the amount of color variation within a clutch was artificially decreased by painting all 3 eggs in the clutch with the same paint shade (Vivid robin-mimetic paint, decreased color variation treatment; DV). We added a third, unmanipulated group (UNM) of nests using data from previous years *sensu* [65]. These nests consisted of clutches containing 2–4 eggs whose colors were not altered but where the eggs were handled and treated otherwise identical to IV and DV nests, and inspected with the same frequency and manner.

Subsequent to manipulating host egg color, we returned the following day, and artificially parasitized nests with plaster-of-Paris model eggs painted a third shade of blue (Fig. 1), also of known rejection rate (58%) from previous experiments [64]. We chose a blue model parasitic egg because behavioral responses to model mimetic cowbird eggs are invariable in our robin population (rejected in 100% of experimental trials, [64]), whereas exposure to the blue model yielded an intermediate rejection rate (58%), with a variable host response [64]. Model ‘parasitic’ eggs mimicked the mass and dimensions of real cowbird eggs, measuring 2.6–3.3 g and with dimensions 21 x 16 mm [66] (as cited in [67]). Model cowbird eggs are known to be rejected at statistically similar rates to real eggs in artificial parasitism studies with American robins [2]. We employed a one-day latency between manipulation and experimental parasitism in order to ensure that no host eggs were rejected as a result of color manipulation (a single pale mimetic host egg was rejected at *n* = 1 nest, out of 27 total nests). Where a 4^th^ egg was laid after manipulation, this was removed in order to keep clutch sizes consistent across IV and DV nests. In most cases, hosts were theoretically able to view these 4^th^ eggs alongside painted eggs for a period of 1–4 hours before removal. Because the degree of variation remained greater in IV than in DV treatments even in nests where a 4^th^ egg was laid, and because hosts were free to view their full unmanipulated clutches in cases where we discovered a clutch already containing 3 eggs, we do not consider that this limited exposure to additional natural eggs has effected the robins’ rejection decisions [65].

We monitored experimental and control nests by returning daily and visually determining the status of the artificial egg, using binoculars and small nest-mirrors as necessary. Eggs were considered rejected if they were not present in the nest on the day following a previous nest-check with the egg still present, except when hatching or predation may have occurred. Model eggs were considered accepted if they remained in the nest for 6 consecutive days (*sensu* [68]), after which nests were emptied, as painting the egg shells inhibits respiratory exchange and thereby prevents proper embryonic development. By disposing of the eggs immediately, we minimized the loss of parental investment and maximize the likelihood of renesting. For each nest we recorded the treatment, date of parasitism (Julian date), nesting stage (laying/incubation), and outcome of parasitism (accept/reject).

Frequency tables of treatment (IV, DV, control) and outcome (accept/reject) data were analyzed using Fisher’s exact test with Monte Carlo simulation based on 2000 replicates. We next evaluated possible effects of nesting stage, clutch size, and Julian date by including these as covariates in fitting a binomial logistic generalized linear mixed model (GLMM) with treatment group and incubation stage as additional possible predictors, and year as a random variable. Experimental parasitism during the laying stage was defined as taking place at any time before or on the day the last egg was laid; at any point beyond it was considered as taking place during the incubation stage. We also fit a binomial logistic generalized linear model (GLM) with variables as listed above, but with year treated as a fixed effect in order to verify that results were not biased by parsing data across only three year levels (*sensu* [69]).

Because our hypotheses predict no effect of manipulating clutch color variation (Table 3), we have also included here a power analysis for our experimental manipulation. All analyses were conducted in R version 2.12.1. This study was conducted in accordance with guidelines for animal care and use as approved by the Institutional Animal Care and Use Committee of Hunter College of the City University of New York (permit number MH 2/13-T3). All manipulations took place on private property and with the explicit consent of the property owner.

## Results

### Avian visual modeling of egg color analysis

Mean quantum receptor catches for natural robin eggs differed significantly more between nests (*n* = 23) than expected based on variation within nests, for four avian single-cone photoreceptors (ultraviolet, UVS; short wavelength, SWS; medium wavelength, MWS; long wavelength, LWS), and for achromatic photoreceptors (Table 4). Mean chromatic discriminability as JNDs, (*n* = 35 comparisons) was greater between nests than would be expected based on variation within nests (Table 4).

**Table 4:** Univariate ANOVA outputs.

Photoreceptor	Mean prop. catch/egg (SE)	Num. df	Den. df	F	*p*
UVS	0.03(0.00)	19.00	14.45	6.25	< 0.005
SWS	0.22(0.00)	19.00	14.17	10.28	< 0.005
MWS	0.37(0.00)	19.00	14.38	8.86	< 0.005
LWS	0.38(0.00)	19.00	15.50	72.08	< 0.005
Achrom	20.78(0.55)	19.00	14.82	10.12	< 0.005
Chrom JNDs	W 0.89(0.53)	1.0	19.98	5.86	< 0.05
	B 2.26(0.19)				

Summary of ANOVA results describing differences in the proportional photoreceptor catches between eggs within versus between unmanipulated host nests. For each photoreceptor type, ‘Mean (SE)’ represents the proportionate receptor catch per egg, and standard error. JNDs values indicate discriminable chromatic difference between two eggs, as perceived by avian visual physiology (see Methods). For JNDs, mean JND values are shown both for within (W) and between (B) nest comparisons. Significant p values for JNDs indicate that mean discriminability was greater between nests than would be expected based on variation within nests. For all measures, there is significantly more variation between nests than within clutches.

### Egg rejection experiment

**Fig 2:**
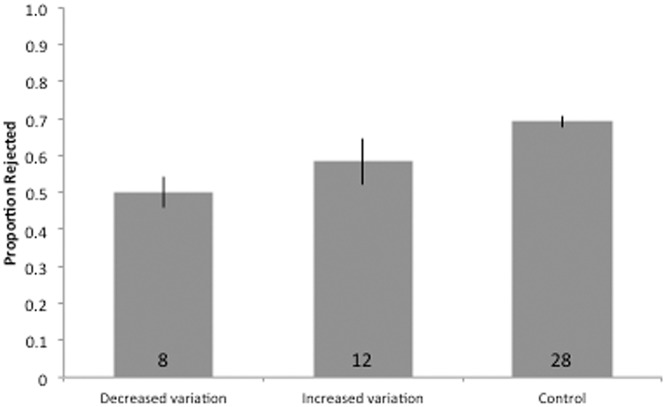
Summary of results of experimental parasitism following the manipulation of clutch contents. Bars represent the rejection rates for parasitic eggs in each experimental group (+ binomial SE estimates). Sample sizes are indicated inside bars.

We found no significant effect of experimentally increasing or decreasing intraclutch color variation on the probability of egg rejection (across all groups Fisher’s exact test, *p* = 0.59; with Monte Carlo simulation, *p* = 0.60; Fig. 2). Likewise, the probability of egg rejection did not differ between IV and DV nests (Fisher’s exact test, *p* = 1; with Monte Carlo simulation, *p* = 1). In a generalized linear mixed model, the likelihood of egg rejection was not statistically predicted by treatment, clutch size, Julian date of artificial parasitism, or incubation stage (binomial logistic regression; see Table 5). Results were qualitatively similar for a model using identical predictors, but where year was treated as a nominal variable and a fixed effect (all *p* > 0.05).

**Table 5:** Summary of binomial GLMM outputs.

Variable	Estimate	Error	z	*p*
Treatment (IV)	-0.15	1.00	-0.15	0.88
Treatment (Con)	0.92	0.86	1.07	0.28
Nesting stage	0.01	0.81	0.01	0.99
Julian date	0.01	0.02	0.45	0.65
Clutch size	-0.31	0.60	-0.51	0.61

Summary of GLMM outputs describing the effects of experimental manipulation, nesting stage (laying versus incubation), and Julian date on the likelihood of the rejection of ‘parasitic’ eggs.

We also conducted a power analysis using the true effect size from the Fisher’s exact test above, as Cramer’s V. Based on Cramer’s V = 0.16 for our actual data set, statistical power = 0.10. To achieve statistical power of 0.8 for this low true effect size, *n* = 396 nest manipulations would be necessary.

## Discussion

Our behavioral experiments showed that the degree of intraclutch color variation had no effect on hosts’ ability to reject parasitic eggs, because the rejection of our model parasitic eggs was independent of intraclutch color variation manipulation. Importantly, in this study we tested for effects of both increasing and decreasing intraclutch color variation. To our knowledge, the effect of decreasing intraclutch color variation has been addressed in only a single prior study, Bán et al. [38], in which investigators manipulated entire great reed warbler (*Acrocephalus arundinaceus*) clutches, dying some eggs or entire clutches with the same color paint or with 3–5 different colors. As predicted for this host of a mimetic brood parasite, the common cuckoo (Table 3), the rejection rates of foreign egg colors in nests with more experimental intraclutch variability were decreased relative to rejection rates in nests with less variability. Notably, relative rejection rates across different color manipulations remained consistent across egg color treatments with different levels of mimicry [70], such that more mimetic blue eggs, for example, were always rejected least often, and less mimetic orange eggs were rejected most often. This implies that hosts use a relative color-based sensory threshold to make decisions whether or not to reject foreign eggs, but responses may be modified by context, as in multiple parasitism [38]. These conclusions highlight the need for further study testing for effects of both increasing and decreasing intraclutch color variation, in order to clearly test for effects on foreign egg rejection.

Some variation in response to experimental manipulation of intraclutch color variation for hosts of mimetic brood parasites (Table 2) may be the result of hosts’ differential use of cognitive mechanisms in the decision to reject foreign eggs (Table 3). Hosts may recognize parasitic eggs using one or more of the following cognitive mechanisms (or additional mechanisms not listed here, e.g. [71,72]): in 1) discordancy-based recognition, hosts use the current nest contents to assess egg identity, and remove egg(s) which are unlike the rest of the clutch [73,74]. In 2) template-based recognition, host females compare clutch contents to a template of their own eggs from memory, with each egg evaluated against the acceptance threshold anchored by this template [28,75]. Template based-recognition allows the discrimination and rejection of foreign eggs when no host eggs are available in the clutch due to multiple parasitism [38]. Finally, in 3) online self-referent phenotype matching, hosts use the current nest contents to assess egg identity, but rejection is not dependent on relative numbers of each egg type within the clutch, as each egg is compared with the hosts known eggs ([38], *sensu* [76]) as identified shortly after laying [12,76].

Even for hosts of mimetic parasites, isolating and testing the specific cognitive mechanisms driving parasitic egg rejection remain challenging. For example, Stevens et al. [39] concluded that tawny-flanked prinias (*Prinia subflava*) use both template-based recognition and discordancy in rejection decision-making, because the rejection of mimetic experimental eggs was mediated in part by the relative numbers of host and parasitic eggs in the clutch. However, they have also shown that prinias rarely reject their own eggs when clutch contents are manipulated such that these are in the minority. Here Stevens et al. [39] may have referred to discordancy without the predictable rejection of the egg in the minority in the clutch, and conflated this with differences in proportion of host versus parasite eggs in the nest. The need to clearly identify the cognitive mechanism underlying egg discrimination highlights the importance for protocols to be better designed to tease apart such closely tied proximate mechanisms, manipulating clutch contents such that specific and different predictions can be made and tested under each cognitive mechanism (Table 3).

However, differential use of cognitive mechanism cannot explain any variation among hosts of non-mimetic parasites because parasitism by a non-mimetic parasite *per se* is predicted to have no effect on the intraclutch color variation or rejection rates for hosts (Table 3). Likewise, investigations of intraclutch color variation and its effect on egg rejection will also be of limited utility in parsing cognitive mechanisms underlying egg rejection in these hosts [38,39,65]. If hosts of non-mimetic parasites utilize a discordancy-based recognition system [70], experimental manipulation of intraclutch variation cannot effect rejection unless clutch contents are modified specifically to make hosts eggs appear similar to parasite eggs, guaranteeing that these eggs are generalizable and recognizable as foreign and allowing the test to focus only on responses elicited by differences in egg number. Likewise, if hosts utilize template-based recognition [77], the characteristics of the existing clutch are again not relevant to decision-making, irrespective of intraclutch variation, unless the recognition template is updated frequently and/or parasitism rates are consistently high. If hosts utilize online self-referent phenotype matching [76], experimental manipulation of intraclutch color variation can only affect rejection rates if hosts are not allowed to view their own eggs at any point prior to manipulation.

In parallel with several other studies focusing on hosts of both mimetic and non-mimetic brood parasitic birds (Table 1, 2), our observations of natural egg coloration in the robins revealed significantly higher perceivable variation between clutches than within clutches, across the sensitivity ranges for all avian photoreceptors. However, increased inter- vs. intraclutch variation in eggshell coloration has been repeatedly detected not only amongst hosts (but see [35]), but also amongst non-hosts of brood parasitic birds [78,79], and thus cannot be a critical test of coevolutionary history with mimetic brood parasitism.

Overall, these results support predictions associated with coevolution between non-mimetic parasitic cowbirds and egg-rejecter robins. In addition, we also demonstrated that for hosts of non-mimetic parasites, parsing the cognitive mechanisms used to make rejection decisions is theoretically challenging. Further research should be focused toward devising new treatments and designs to tease apart the cognitive mechanisms driving parasitic egg rejection (e.g. [70]), particularly in hosts of non-mimetic parasites, where testing for effects of both increasing and decreasing intraclutch color variation does not provide informative tests between alternative cognitive mechanisms (Table 3).

## Supporting Information

S1 Dataset Behavioral outcomes of artificial parasitism. (TXT)

S2 Dataset Color spectra of unmanipulated robin eggs. (TXT)
